# Recurrent Radiation-Induced Angiosarcoma of the Breast Without Distant Metastasis: A Case Report

**DOI:** 10.7759/cureus.106740

**Published:** 2026-04-09

**Authors:** Ivan Bivolarski, Martin Dimitrov

**Affiliations:** 1 Medical Oncology, Chronotherapy and Cancer Research Unit, Integrated Oncology Centre Burgas, Burgas, BGR; 2 Visceral Surgery, Klinik für Allgemeine, Gefäß- und Viszerale Chirurgie, Kreiskrankenhaus Greiz, Greiz, DEU

**Keywords:** chronotherapy, exemestane, luminal like-breast cancer, pazopanib maintenance, radiation-induced sarcoma, systemic inflammation

## Abstract

Radiation-induced sarcomas represent a rare but aggressive complication following breast cancer treatment, often associated with significant biological heterogeneity and limited therapeutic options. Their clinical course is frequently characterized by high recurrence rates and limited responsiveness to conventional therapies.

We report a case of a 76-year-old female with a history of left-sided breast cancer treated in 2017, who subsequently developed a high-grade radiation-induced soft tissue sarcoma of the chest wall. The patient underwent multiple surgical resections due to recurrent disease. Systemic treatment included exemestane as endocrine therapy and pazopanib as antiangiogenic targeted therapy, both administered according to a consistent daily schedule.

Laboratory findings demonstrated a relatively stable neutrophil-to-lymphocyte ratio of approximately 3.0-3.5 in the context of a persistent inflammatory profile. Despite aggressive tumor biology and multiple comorbidities, the patient maintained a relatively stable clinical condition without evidence of distant metastasis.

An additional observation in this case was the consistent daily timing of systemic therapy administration. While not formally evaluated, this pattern may represent a potentially relevant aspect of treatment delivery and could warrant further investigation.

## Introduction

Radiation-induced sarcomas are rare but highly aggressive malignancies that may arise as late complications of radiotherapy in patients treated for breast cancer. Although their incidence remains low, reported rates range between 0.03% and 0.2%, and their clinical course is often characterized by rapid progression, high recurrence rates, and limited responsiveness to conventional therapies. Reported five-year overall survival rates remain poor, typically ranging between 20% and 50%, reflecting the challenging prognosis and complex management of these tumors [1,2]. The biological heterogeneity of radiation-associated sarcomas further complicates therapeutic decision-making. Treatment strategies generally include surgical resection, systemic therapies such as chemotherapy or targeted agents (e.g., pazopanib), and, in selected cases, endocrine therapy when hormone receptor expression is present.

In parallel, increasing attention has been directed toward the role of systemic inflammation and tumor-host interactions in cancer progression [3,4]. Biomarkers such as the neutrophil-to-lymphocyte ratio (NLR) have demonstrated prognostic significance across a wide range of solid tumors, reflecting underlying immune and inflammatory processes [5]. However, these markers are typically interpreted as static values, without accounting for potential temporal variability.

Accumulating evidence suggests that key physiological processes, including immune function, hormonal signaling, and angiogenesis, exhibit circadian variation. These fluctuations may influence immune cell activity, cytokine production, endocrine pathways, and tumor microenvironment dynamics. Angiogenic signaling pathways targeted by tyrosine kinase inhibitors such as pazopanib, as well as endocrine pathways modulated by aromatase inhibitors such as exemestane, may be subject to temporal regulation [6,7]. While these observations provide a biological rationale, direct clinical evidence linking treatment timing to outcomes for these specific agents remains limited.

Here, we present a case of a patient with breast cancer and subsequent radiation-induced sarcoma treated with a combination of exemestane and pazopanib administered according to a consistent daily schedule. This report explores the potential relevance of treatment timing and systemic inflammatory markers in this context, as a hypothesis-generating observation, without implying causality.

## Case presentation

A 76-year-old female with a history of left-sided breast cancer was initially diagnosed in August 2017 with invasive ductal carcinoma of the left breast (pT1cN0M0, grade 3; tumor size = 1.5 cm). She underwent breast-conserving surgery (quadrantectomy) with axillary lymph node dissection, followed by adjuvant chemotherapy and radiotherapy.

Immunohistochemical analysis demonstrated negative HER2 expression (score 0) and positive hormonal receptor status, with an estrogen receptor (ER) score of 5 and a progesterone receptor (PR) score of 4. Between September 13 and October 18, 2017, she received adjuvant radiotherapy to the left breast using 6 MeV photon beams, with a total dose of 50 Gy delivered in 2 Gy fractions. The treatment was well tolerated, with no significant acute complications.

Postoperative follow-up was unremarkable. A whole-body bone scintigraphy performed on February 15, 2018, demonstrated no evidence of metastatic disease, with findings limited to degenerative skeletal changes.

In February 2023, approximately six years after the initial diagnosis, the patient developed a local recurrence and underwent repeat quadrantectomy. Histopathological examination again confirmed invasive ductal carcinoma.

Shortly thereafter, in March 2023, cutaneous changes developed within the previously irradiated breast region. Histopathological evaluation of skin and subcutaneous tissue revealed a malignant vascular neoplasm composed of anastomosing vascular channels lined by atypical endothelial cells with hyperchromatic nuclei, embedded in fibrotic stroma with areas of hemorrhage. Immunohistochemistry showed strong ERG positivity, confirming endothelial differentiation, while HHV8 (LANA1) was negative, excluding Kaposi sarcoma. These findings established the diagnosis of post-radiation cutaneous angiosarcoma.

**Figure 1:**
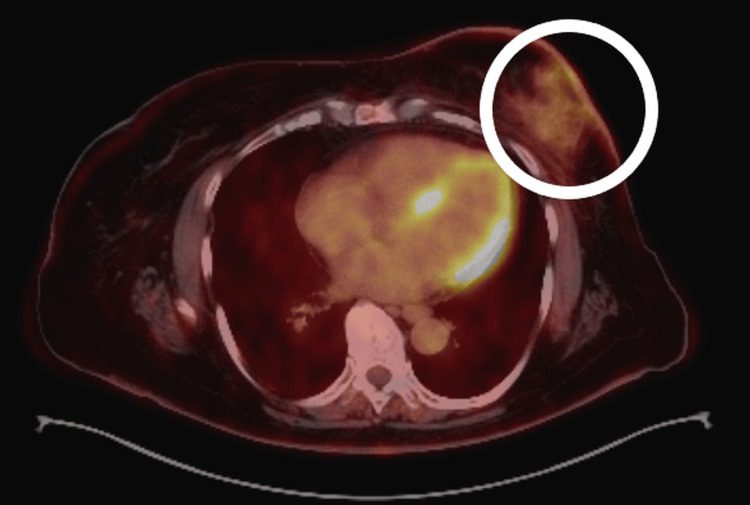
Axial PET/CT image (April 2023) demonstrating focal fluorodeoxyglucose uptake in the left breast/chest wall (circled), without evidence of distant metastatic disease.

A whole-body PET/CT scan performed in April 2023 demonstrated diffuse low-to-moderate metabolic activity in the left chest wall (maximum standardized uptake value (SUVmax) = 3.21), interpreted as postoperative or inflammatory in origin. Mildly metabolically active right axillary lymph nodes (SUVmax = 2.15) and a right inguinal lymph node (SUVmax = 4.23) were considered reactive. No evidence of distant metastatic disease was identified (Figure 1).

**Figure 2:**
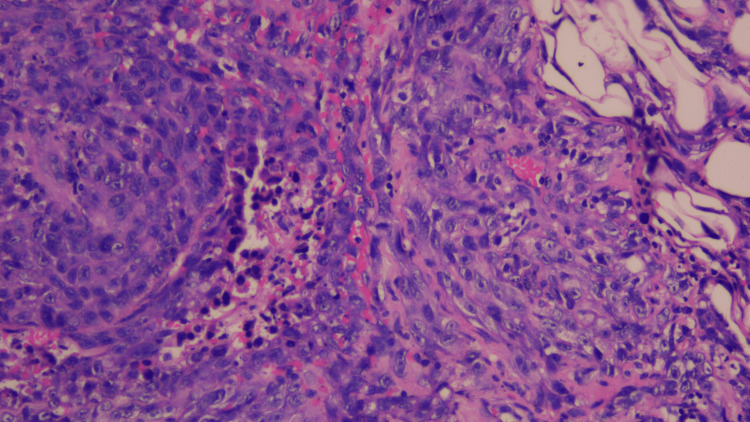
Histopathological appearance of radiation-induced angiosarcoma. High-power hematoxylin and eosin (H&E) stain demonstrating a malignant vascular neoplasm composed of atypical endothelial cells with marked nuclear pleomorphism and hyperchromasia. The tumor cells are arranged in irregular, anastomosing vascular channels within a fibrotic stroma.

On April 20, 2023, a new cutaneous lesion within the previously irradiated field was biopsied, confirming a sarcoma arising in the irradiated area. A multidisciplinary tumor board recommended radical surgical management (Figure 2).

In June 2023, the patient underwent a left radical mastectomy. The postoperative course was uneventful, and she was discharged in stable condition on June 12, 2023.

Despite surgical management, the disease demonstrated a locally aggressive course, with multiple recurrences requiring repeated interventions. In November 2023, PET/CT imaging revealed findings suggestive of local recurrence in the left chest wall (SUVmax = 5.4), without evidence of distant metastases. On November 21, 2023, the patient underwent electroexcision of a recurrent tumor with macroscopically clear margins, followed by reconstruction using a latissimus dorsi flap. The postoperative course was complicated by lymphatic leakage (seroma).

**Figure 3:**
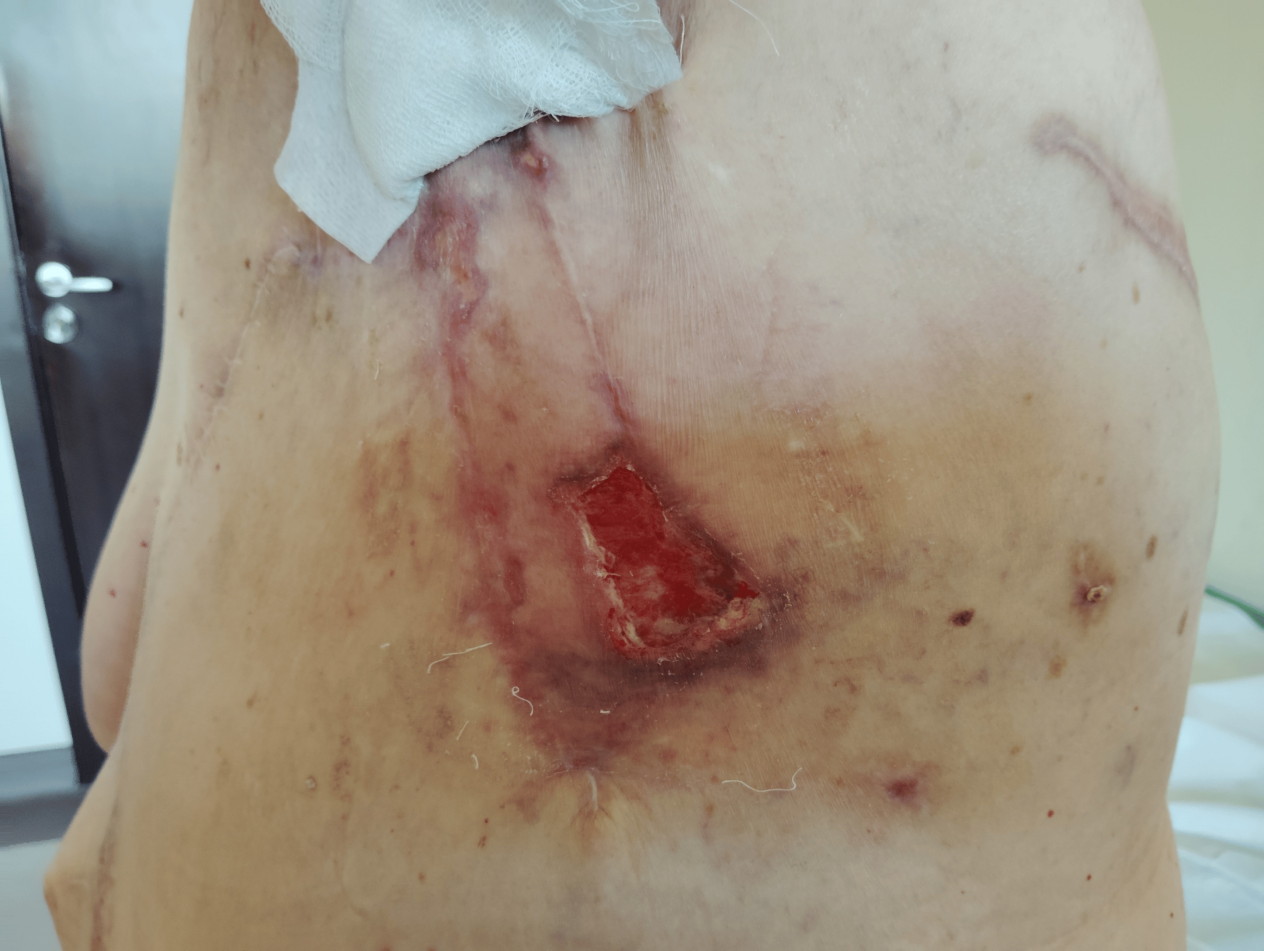
Clinical appearance of chest wall lesion. Clinical photograph demonstrating an ulcerated cutaneous lesion within the previously irradiated left chest wall. The lesion is characterized by irregular margins, surrounding erythema, and violaceous discoloration, consistent with recurrent angiosarcoma in a post-surgical field.

In January 2024, histopathological examination confirmed high-grade angiosarcoma. Throughout 2024, the patient required multiple additional surgical procedures due to recurrent disease and wound-related complications, including interventions in April, June, and November. In October 2024, a non-healing lesion developed in the left chest wall, necessitating further surgical management. In March 2025, she underwent additional reconstructive surgery (Figure 3).

In February 2024, a multidisciplinary decision was made to initiate systemic therapy with pazopanib in combination with exemestane, which was commenced in March 2024. Both agents were administered orally on a consistent daily schedule, with minimal variation in administration timing.

**Figure 4:**
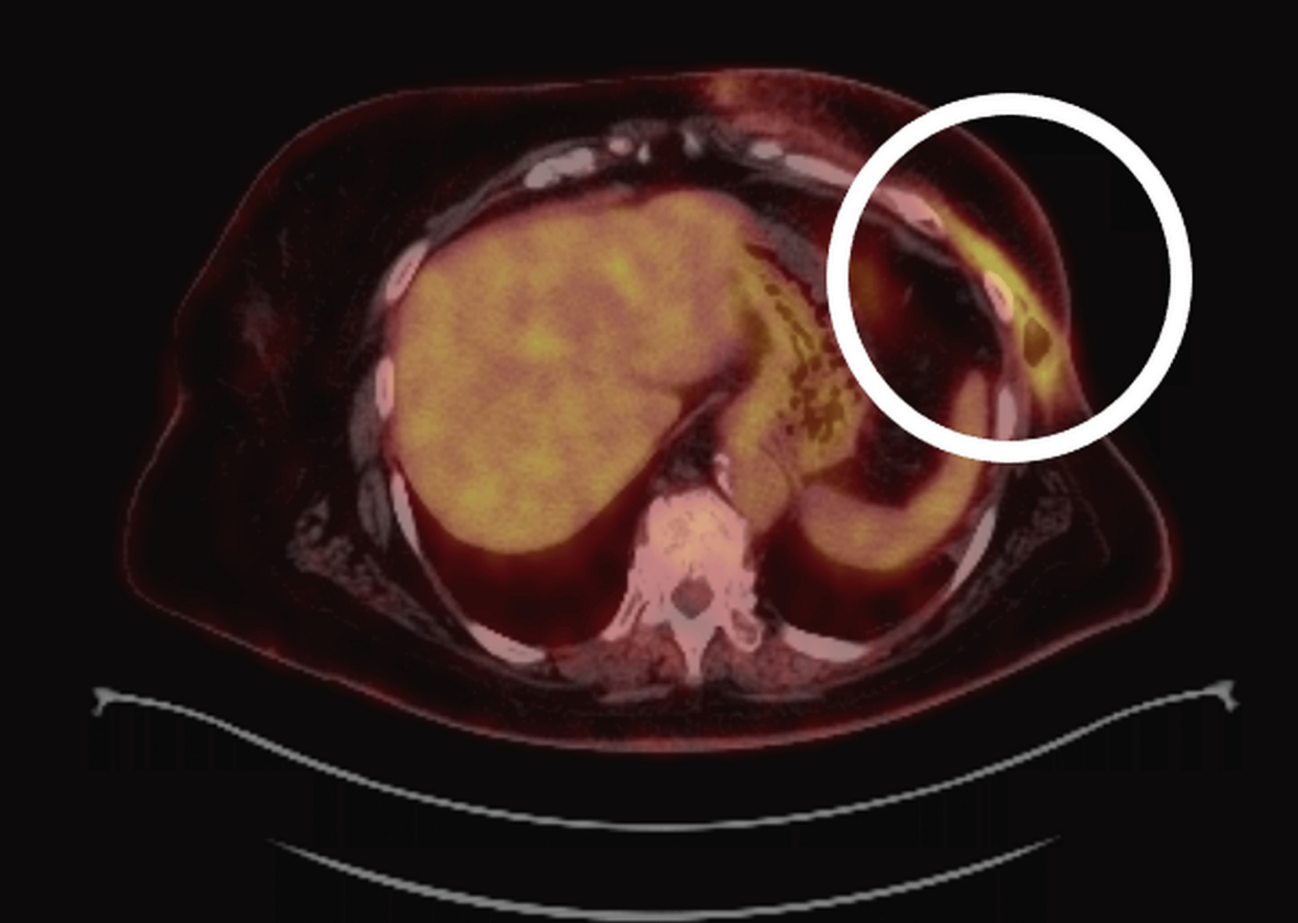
PET/CT imaging during systemic therapy (July 2024). Axial PET/CT image demonstrating focal increased fluorodeoxyglucose uptake in the left anterior chest wall (circled), consistent with persistent localized disease. The metabolic activity is increased compared to prior imaging (maximum standardized uptake value (SUVmax) = approximately 5.7), without evidence of distant metastatic spread.

A PET/CT scan performed in July 2024 demonstrated metabolically active soft tissue lesions in the left anterior chest wall (SUVmax = 5.77 vs. 5.40 previously), consistent with persistent local disease. No evidence of systemic dissemination was observed (Figure 4).

**Figure 5:**
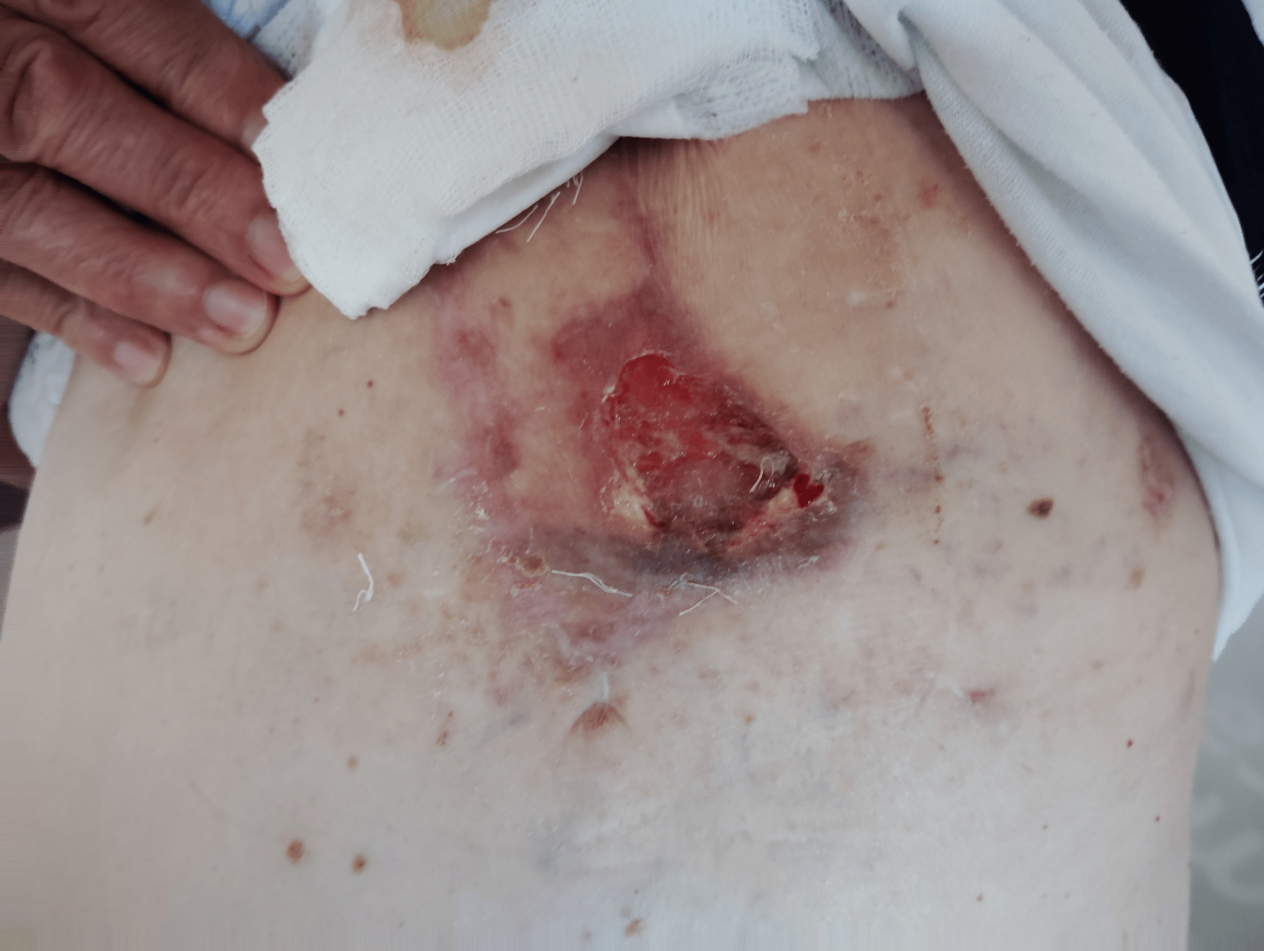
Clinical appearance during systemic therapy. Clinical photograph demonstrating a persistent ulcerated lesion in the left chest wall following initiation of systemic therapy with pazopanib. The lesion shows irregular borders, areas of necrosis, and surrounding erythematous to violaceous discoloration within the previously irradiated and surgically treated field.

Clinically, the patient reported partial improvement in local symptoms, including reduced pain and decreased exudation from the lesion. On physical examination, there was a tendency toward stabilization of the ulcerated area, without signs of rapid progression (Figure 5).

**Figure 6:**
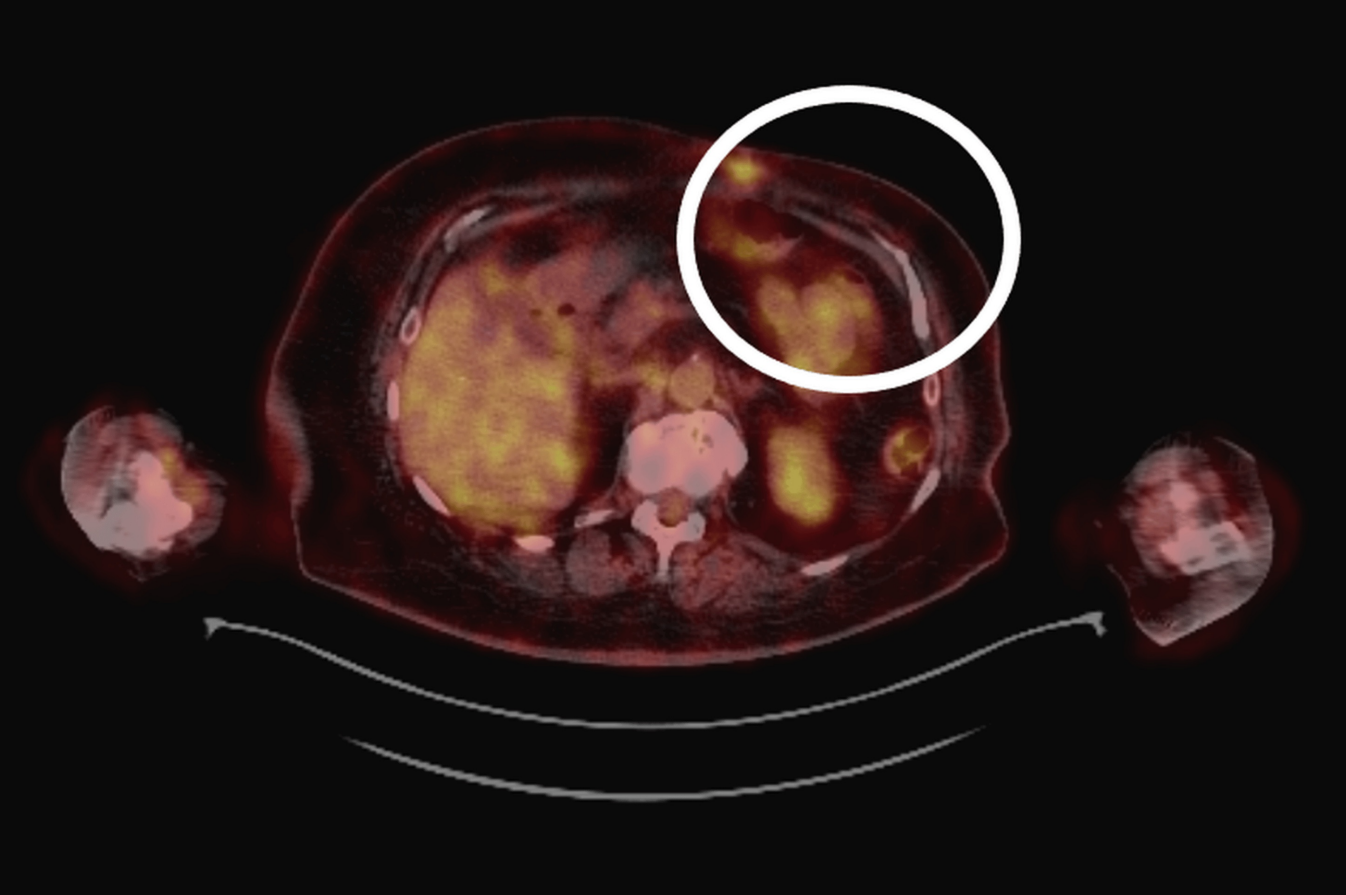
PET/CT imaging (May 2025). Axial PET/CT image demonstrating multiple areas of increased fluorodeoxyglucose uptake in the left chest wall (circled), consistent with persistent metabolically active disease. A focal area of increased uptake is also noted in the liver region without a clear structural correlation on CT. Subsequent imaging did not confirm hepatic involvement. Overall, no definitive evidence of distant metastatic disease is identified.

In May 2025, PET/CT imaging demonstrated several metabolically active foci in the left chest wall (SUVmax = 3.68), likely reflecting postoperative or reactive changes. A focal area of increased metabolic activity was also observed in liver segment VI (SUVmax = 5.79), without corresponding structural findings on CT. Subsequent imaging, including abdominal MRI performed in June 2025, demonstrated no evidence of hepatic lesions (Figure 6).

**Figure 7:**
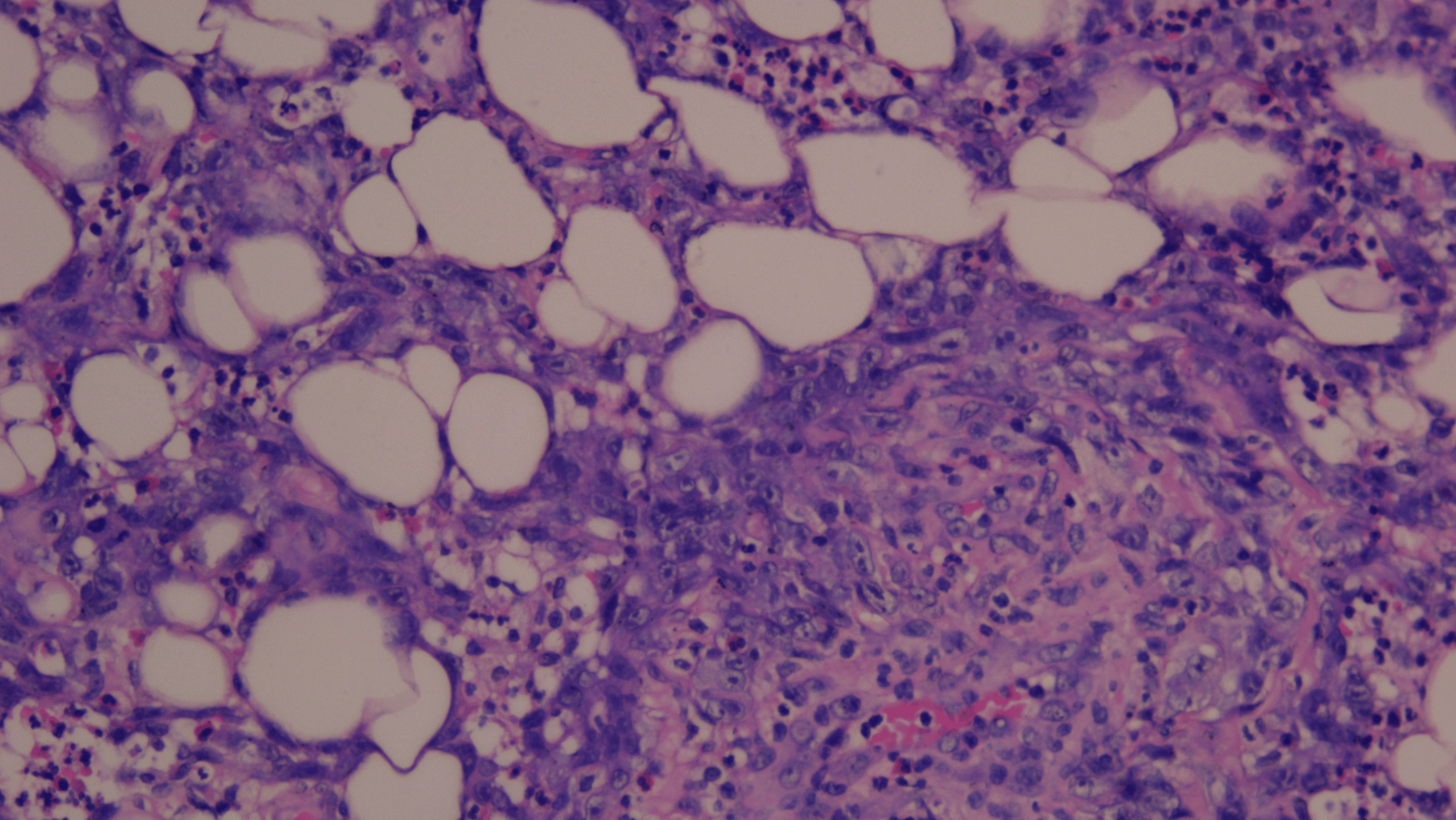
Histopathological features demonstrating tumor infiltration (2025). High-power hematoxylin and eosin (H&E) stain demonstrating infiltration of atypical endothelial tumor cells into surrounding adipose tissue. Irregular vascular channels and scattered pleomorphic cells are observed between adipocytes, supporting an infiltrative growth pattern.

In July 2025, pazopanib was temporarily discontinued for 20 days to facilitate wound healing and was subsequently resumed in August 2025. A repeat biopsy performed in September 2025 confirmed the presence of persistent poorly differentiated tumor tissue (Figure 7).

**Figure 8:**
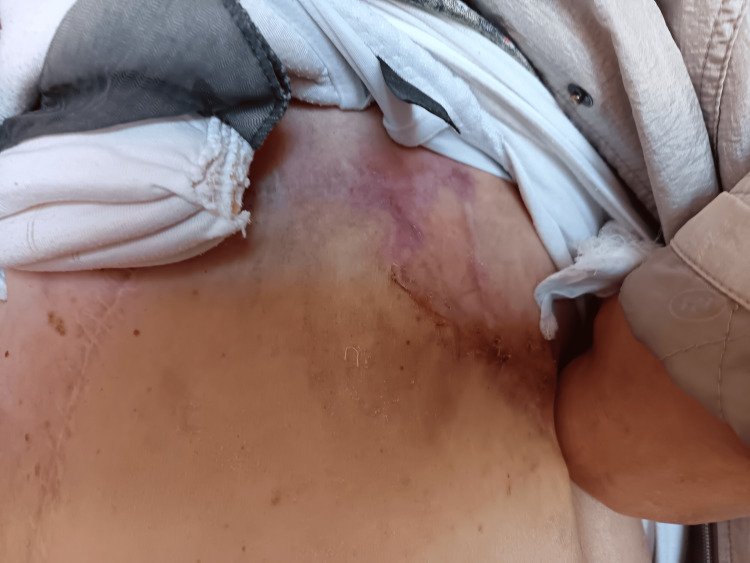
Clinical appearance during follow-up. Clinical photograph demonstrating partial regression and stabilization of the lesion in the left chest wall during ongoing systemic therapy. The previously ulcerated area appears reduced, with residual discoloration and post-treatment changes within the irradiated field.

Following prolonged local management and continuation of systemic therapy, gradual wound healing was observed, ultimately leading to closure of the lesion. At follow-up, the previously ulcerated area appeared fully epithelialized, with no active exudation or signs of acute local progression (Figure 8).

**Figure 9:**
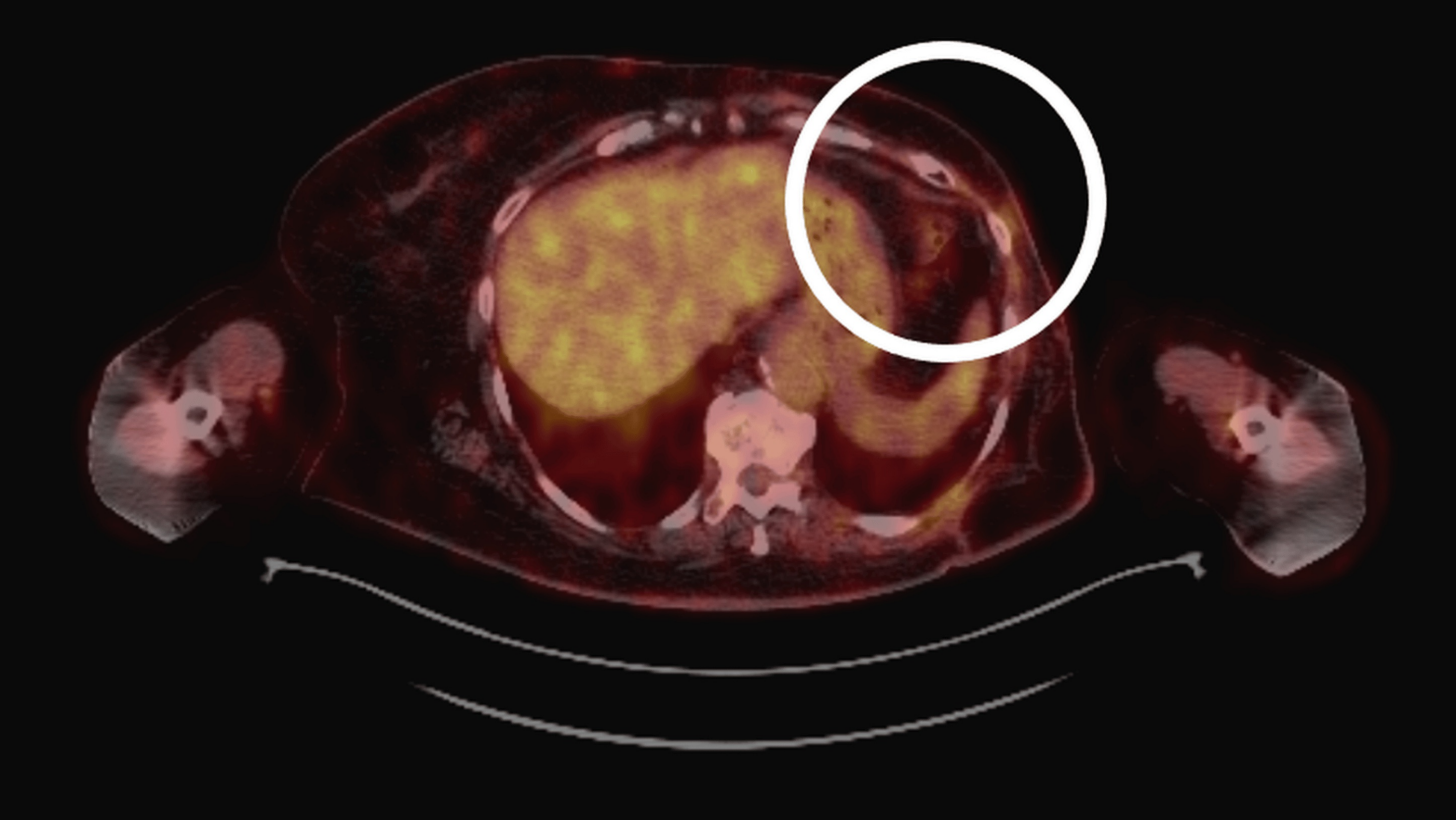
Follow-up PET/CT imaging (December 2025). Axial PET/CT image demonstrating persistent focal fluorodeoxyglucose uptake in the left anterior chest wall (circled), consistent with ongoing metabolically active localized disease. No evidence of lymph node or distant organ involvement is identified.

Serial imaging demonstrated a dynamic yet consistently localized disease pattern. A PET/CT scan performed on December 18, 2025, revealed persistent metabolically active soft tissue in the left anterior chest wall (SUVmax = 3.92), without evidence of lymph node or distant organ involvement (Figure 9).

Across multiple imaging modalities and time points, the disease remained consistently localized, without evidence of distant dissemination.

The latency period between radiotherapy (2017) and the development of angiosarcoma (2023) was approximately six years.

Despite aggressive tumor biology, repeated local recurrences, and multiple surgical interventions, the patient has maintained a relatively stable clinical condition under ongoing treatment with pazopanib and exemestane as of March 2026.

## Discussion

Radiation-induced sarcomas represent a distinct clinical entity characterized by aggressive behavior, high recurrence rates, and limited therapeutic options, particularly in elderly patients with multiple prior interventions [8,9]. In the present case, the coexistence of a high-grade sarcoma in the setting of prior breast cancer underscores the complexity of tumor biology, including the potential contribution of therapy-induced mutagenesis and clonal evolution. This biological heterogeneity may contribute to variable treatment responses and complicates clinical interpretation.

Systemic inflammation and immune regulation are increasingly recognized as relevant factors in cancer progression. Elevated inflammatory markers, including CRP and NLR, have been associated with adverse outcomes across multiple malignancies [10]. In this patient, NLR values remained moderately elevated (~3.0-3.5) over time, consistent with a chronic inflammatory state. However, given the descriptive nature of this observation, no direct relationship between inflammatory markers and the clinical course can be established.

Time-dependent biological variability has been described across several physiological systems, including immune function, endocrine signaling, and angiogenesis [11-13]. These processes may influence tumor-host interactions through mechanisms such as immune cell trafficking, cytokine release, and hormonal regulation. Pathways targeted by tyrosine kinase inhibitors such as pazopanib, as well as endocrine pathways modulated by aromatase inhibitors such as exemestane, may also be subject to temporal variation [13,14]. However, clinical evidence directly linking such variability to treatment outcomes for these agents remains limited.

In the present case, the timing of exemestane and pazopanib administration remained consistent throughout the treatment period. This observation is reported descriptively. Although treatment timing has been proposed as a potential modifier of therapeutic effects, it was not formally evaluated in this case, and no causal relationship can be inferred.

Pharmacokinetic variability may represent another relevant consideration. Factors such as hepatic metabolism, drug absorption, and clearance may vary over time, potentially affecting systemic drug exposure [15,16]. This may be particularly relevant for orally administered agents such as pazopanib. However, no pharmacokinetic measurements were performed in this case, and these considerations remain theoretical.

Overall, this case illustrates the complexity of interpreting clinical stability in radiation-induced sarcoma. Notably, despite aggressive tumor biology and repeated local recurrences, the disease remained consistently localized, with preservation of a relatively stable clinical condition under ongoing systemic therapy. The observations presented should be regarded as descriptive and hypothesis-generating. Further studies are required to evaluate the potential role of treatment timing and temporal biological variability in oncologic outcomes.

## Conclusions

This case illustrates the clinical complexity of radiation-induced sarcomas following breast cancer, particularly in the context of prior multimodal therapy and persistent systemic inflammation. The coexistence of endocrine-related and angiogenesis-related mechanisms reflects the heterogeneous biological behavior of these tumors.

The relatively stable clinical course observed in this patient, despite high-risk pathological features, represents an observational finding. The consistent timing of combined exemestane and pazopanib administration is noted as part of the treatment course. While biological processes relevant to tumor progression and treatment response may exhibit temporal variability, no causal relationship can be established based on a single case. This observation should be considered hypothesis-generating and requires further investigation in larger studies.
